# Supplementary material for: Mechanisms governing the pioneering and redistribution capabilities of the non-classical pioneer PU.1
Source: Nat Commun. 2020 Jan 21;11:402. doi: 10.1038/s41467-019-13960-2 (PMC6972792; doi:10.1038/s41467-019-13960-2)
Supplement: Supplementary file 7 — Source data [file 41467_2019_13960_MOESM7_ESM.zip › Source_Data/Figure6/Figure6B_MotifScanOutput/ETS-specific/homerResults/motif16.similar.html]

motif16

## Information for motif16

T
C
A
G
G
T
A
C
G
A
C
T
T
C
A
G
C
G
A
T
G
A
T
C
C
G
T
A
  
Reverse Opposite:  

G
C
A
T
C
T
A
G
C
G
T
A
A
G
T
C
C
T
G
A
C
A
T
G
A
G
T
C
  

|  |  |
| --- | --- |
| p-value: | 1e-66 |
| log p-value: | -1.524e+02 |
| Information Content per bp: | 1.775 |
| Number of Target Sequences with motif | 3187.0 |
| Percentage of Target Sequences with motif | 41.63% |
| Number of Background Sequences with motif | 12660.0 |
| Percentage of Background Sequences with motif | 31.37% |
| Average Position of motif in Targets | 98.7 +/- 57.0bp |
| Average Position of motif in Background | 100.6 +/- 62.2bp |
| Strand Bias (log2 ratio + to - strand density) | -0.0 |
| Multiplicity (# of sites on avg that occur together) | 1.26 |
| Motif File: | file (matrix) reverse opposite |

### Similar de novo motifs found

|  |  |  |  |  |  |  |  |
| --- | --- | --- | --- | --- | --- | --- | --- |
| Rank | Match Score | Redundant Motif | P-value | log P-value | % of Targets | % of Background | Motif file |
| 1 | 0.935 | A T C G G T A C A C G T C T A G G C A T G A T C C G T A C T A G | 1e-50 | -117.098795 | 25.41% | 17.81% | motif file (matrix) |
| 2 | 0.808 | G A T C C G T A C T A G G T A C C G A T C T A G G A C T T G A C C G T A | 1e-41 | -96.130975 | 14.38% | 9.07% | motif file (matrix) |
